# Targeting PD-1/PD-L-1 immune checkpoint inhibition for cancer immunotherapy: success and challenges

**DOI:** 10.3389/fimmu.2024.1383456

**Published:** 2024-04-10

**Authors:** Sadique A. Javed, Asim Najmi, Waquar Ahsan, Khalid Zoghebi

**Affiliations:** Department of Pharmaceutical Chemistry, College of Pharmacy, Jazan University, Jazan, Saudi Arabia

**Keywords:** cancer immunotherapy, immune checkpoint, monoclonal antibodies, small molecule inhibitors, PD-1/PD-L-1

## Abstract

The programmed death-1 receptor (PD-1) acts as a T-cell brake, and its interaction with ligand-1 (PD-L-1) interferes with signal transduction of the T-cell receptor. This leads to suppression of T-cell survival, proliferation, and activity in the tumor microenvironment resulting in compromised anticancer immunity. PD-1/PD-L-1 interaction blockade shown remarkable clinical success in various cancer immunotherapies. To date, most PD-1/PD-L-1 blockers approved for clinical use are monoclonal antibodies (mAbs); however, their therapeutic use are limited owing to poor clinical responses in a proportion of patients. mAbs also displayed low tumor penetration, steep production costs, and incidences of immune-related side effects. This strongly indicates the importance of developing novel inhibitors as cancer immunotherapeutic agents. Recently, advancements in the small molecule-based inhibitors (SMIs) that directly block the PD-1/PD-L-1 axis gained attention from the scientific community involved in cancer research. SMIs demonstrated certain advantages over mAbs, including longer half-lives, low cost, greater cell penetration, and possibility of oral administration. Currently, several SMIs are in development pipeline as potential therapeutics for cancer immunotherapy. To develop new SMIs, a wide range of structural scaffolds have been explored with excellent outcomes; biphenyl-based scaffolds are most studied. In this review, we analyzed the development of mAbs and SMIs targeting PD-1/PD-L-1 axis for cancer treatment. Altogether, the present review delves into the problems related to mAbs use and a detailed discussion on the development and current status of SMIs. This article may provide a comprehensive guide to medicinal chemists regarding the potential structural scaffolds required for PD-1/PD-L-1 interaction inhibition.

## 1 Introduction

Programmed death-1receptor (PD-1) was first identified in the year 1992 initially as an apoptosis-associated gene (1). Later, PD-1 expression through T- and B-lymphocyte antigen receptor signaling was reported (1, 2). PD-1 is produced in activated T-cells and functions as an inhibitory receptor inhibiting the immunological responses (3, 4). On the other hand, cancer and antigen-presenting cells in the tumor microenvironment (TME) produce PD-1 ligands including PD-L-1 (B7-H1) and PD-L-2 (B7-DC), which, impair anticancer immunity when bind to the PD-1 receptor (5–7). Cancer immunotherapy has been revolutionized by the discovery that PD-1 and PD-L-1 are the novel targets for eliciting T-cell antitumor responses and through the development of antibodies that can block the PD-1/PD-L-1 axis (8–10).

Preclinical and clinical investigations have indicated that in comparison to anti-CLTA-4 inhibitors, antibodies inhibiting PD-1 and its ligands display a broader spectrum of anticancer efficacy and lower toxicity. The outcomes significantly enhanced the clinical trials and US FDA approval rate of PD-1/PD-L-1 blocking molecules (11). These development further accelerated the use of PD-1 based immunotherapy for various types of cancers in outpatient settings. Currently, a number of antibodies are clinically being used, and several small compounds are in the development pipeline as potential medicines for cancer treatment; however, in most cases transient or no responses have been observed following monotherapy with currently available agents and durable clinical responses are observed only in a small fraction of cases. Consequently, to produce durable anticancer responses, immunotherapy based on PD-1/PD-L1 inhibition is used in conjunction with other available therapeutic options, including CTLA-4 blockade, cancer vaccines, target-based small molecule inhibitors (SMIs) and agonist antibodies (12).

The current development of PD-1/PD-L1 blocking agents indicated that this pathway is a potential target for inducing anticancer immunity. Consequently, during the last few years, this has generated tremendous interest in the scientific fraternity involved in drug discovery research for exploring the target to achieve therapeutic success in cancer immunotherapy. However, important information regarding in-depth mechanism by which PD-1 signaling is involved in cancer mediated immune-suppression, with the aim of identifying new targets for drug action and exploitation of the target to design and develop safe and effective medication, is warranted.

## 2 PD-1/PD-L1 expression

**Figure 1:**
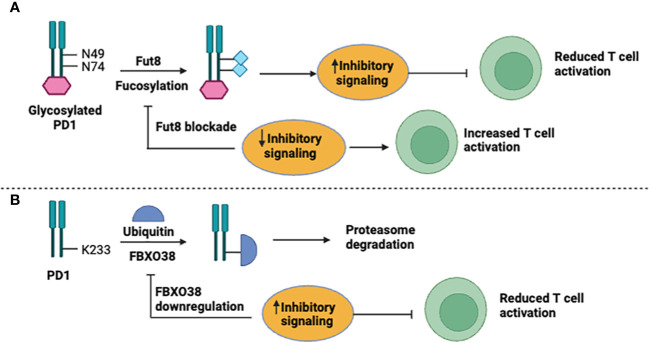
Mechanisms for PD-1 expression. **(A)** Fucosylation and **(B)** Ubiquitnation.

In recent years, the expression of PD-1 and PD-L1 has been extensively studied and found to occur in both the adaptive and innate immune systems (13, 14). Investigations indicated that the PD-1/PD-L1 expression has been regulated by transcriptional and post-translational mechanisms (**Figure 1**). It was reported that fucosylation of glycosylated PD-1 at N49 and N74 residues by a core Fut8 fucosyltransferase resulted in increased PD-1 expression at the cell-surface, resulting in the conduction of inhibitory signals (15). Genetic depletion or pharmacological blockade of Fut8 fucosylase reduced inhibitory signals by decreasing PD-1 fucosylation expression that subsequently resulted in significant *in vivo* antitumor responses through activation of T-cells (**Figure 1A**). Though, the precise mechanisms of the structural and functional changes in PD-1 need to be elucidated. Another study indicated that E3 ligase FBXO38 regulates PD-1 expression; it facilitates K48-associated polyubiquitination (UQ) of PD-1 at the K233 position, leading to proteasomal degradation of PD-1. Downregulation or genetic ablation of FBXO38 enhances PD-1 expression, which in turn increases inhibitory signaling and suppresses T cells (16) (**Figure 1B**).

**Figure 2:**
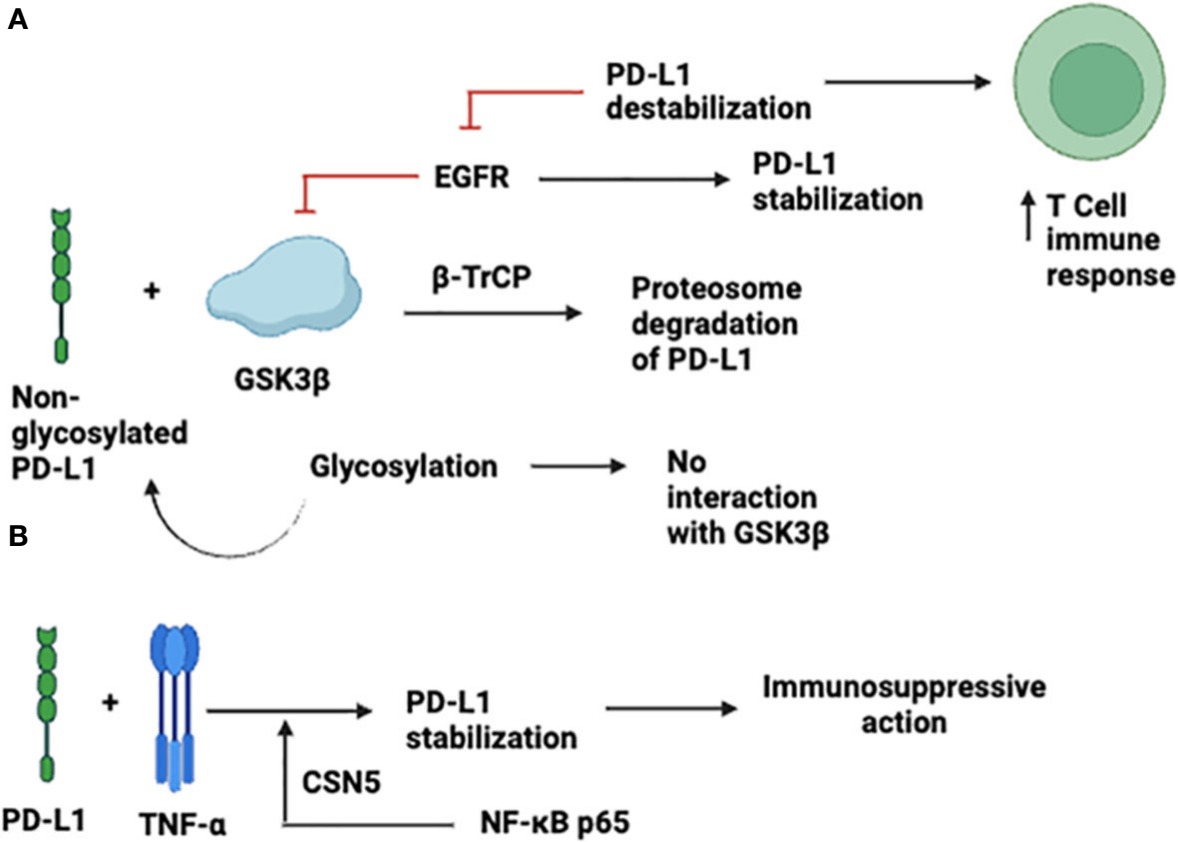
Expression and Stabilization of PD-L-1. **(A)** through inactivation of GSK3β; **(B)** TNF-α -mediated expression.

Glycosylation and ubiquitination strictly regulate the stabilization, expression, and immunosuppressive effects of PD-L-1 (17). The glycogen synthase kinase-3β (GSK3β) non-glycosylated PD-L-1 interact and cause phosphorylation-dependent proteasomal degradation of PD-L1 by β-transducin repeat-containing protein; however, the GSK3β interaction is antagonized by glycosylation (**Figure 2**). Moreover, it has been documented that in basal-like breast cancer, epidermal growth factor (EGF) stabilizes PD-L1 through inactivation of GSK3β. PD-L-1 destabilization by gefitinib via inhibition of EGF signaling boosts anti-tumor T-cell immunity and enhances the effectiveness of cancer immune-therapy (**Figure 2A**) (17). TNF-α is a primary factor in immune suppression of tumor cells against T-cells through PD-L-1 stabilization. TNF-α mediates the stabilization of PD-L in tumor cells, which prevents PD-L-1 from being ubiquitinated and degraded, necessitating NF-κB p65-induced COP9 signalosome 5 (CSN5). This was further demonstrated by the suppression of CSN5-mediated reduction in PD-L-1 expression in cancer cells, which increased the reactivity of the cells to anti-CTLA4 treatment (18) (**Figure 2B**).

PD-L-1 needs to be glycosylated for interaction with PD-1, and it is also important to maintain the binding capacity of soluble PD-L-1 (19); however, glycosylation of PD-1 is not necessary for this interaction, as structural investigations indicated that the PD-1/PD-L1-binding interface is situated away from PD-1 glycosylation locations, indicating that this interaction is not directly influenced by PD-1 glycosylation (20). Therapeutic antibodies that inhibit PD-1, such as pembrolizumab and nivolumab, can bind to proteins without glycosylation of PD-1 (21, 22). The above observations indicated that ubiquitination and glycosylation processes are strongly linked to PD-1/PD-L-1 expression and provide an important target for new therapeutic approach for improving the effectiveness of cancer immunotherapy.

Therefore, understanding the fundamental processes that underlie the regulation of PD-L-1 in tumor cells may assist in the development of immune checkpoint blockade (ICB) treatment. In normal tissues, PD-L-1 expression is tightly regulated at the transcriptional and posttranslational levels in response to both internal and external stressors in order to reduce tissue inflammation and support maintaining homeostasis (23, 24). On the other hand, the abnormal expression of PD-L-1 is utilized by the tumor cells to avoid immune surveillance (25). Posttranslational changes, including ubiquitination (18), glycosylation (17), phosphorylation (26), acetylation (27), and palmitoylation (28), have been shown recently to influence the stability of PD-L-1 and ultimately the cancer immune surveillance is affected. PD-L-1 stability and nuclear localization can be decreased by inhibitors of CSN5 and HDAC2, which are known to be the post-translational modification regulators of PD-L-1. This makes them useful tools for combinational therapy when used in conjunction with immune checkpoint therapy (23, 27). With the exception of glycosylation, the process underlying the post-translational modification of PD-L-1 within the ER is still not well understood (24, 29). Recent studies have shown that ER homeostasis depends critically on the ER-resident UFMylation (30–32).

The uncharacterized protein CMTM6 was shown to be a PD-L-1 regulator on certain cancer cell surfaces using genome-wide CRISPR-Cas9 screening, and further research revealed its regulatory function on the PD-L-1. CMTM6 maintains its expression on cell surface by binding to PD-L1. CMTM6 has been identified as a critical PD-L-1 stabilizer in a number of cancer types, such as thyroid, colorectal, pancreatic, and non-small cell lung cancers (NSCLC). It was discovered that CMTM6 binds to PD-L-1 on plasma membrane and recycled endosomes, protecting PD-L-1 from lysosome dependent degradation (33). Conversely, it was shown that cancer cells expressed less PD-L-1, which may be partly restored by STUB1 E3 ubiquitin ligase deletion, responsible for the PD-L degradation and downregulation of its expression. Moreover, in case of modified genetic screening in haploid cells deficient in CMTM6, CMTM4 was found to be a complementary regulator of PD-L-1 expression (34). The suppression of tumor-specific T-cell activity was significantly reduced by CMTM6 depletion through PD-L1 destabilization in tumor cells. Additionally, when CMTM6 expression is disrupted, there is a decrease in both constitutive and INF-α-induced protein expression of PD-L-1 (33, 34).

## 3 PD-1/ligands interaction

**Figure 3:**
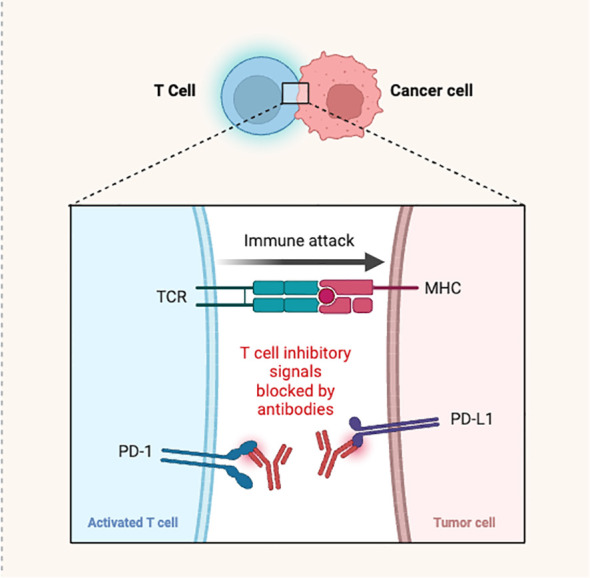
Antibodies blocking the PD-1/PD-L-1 interaction causing T-cell activation and immune attack.

PD-1 is a specific receptor ligated by PD-L-1 ligand, resulting in a negative T-cell receptor (TCR) regulation. It was previously discovered that PD-1/PD-L-1 interaction reduces cytokine production and TCR-mediated proliferation. Compared to normal tissue cells, which showed minimal PD-L-1 surface expression, significantly greater expression was observed in human cancer cells (35). Generally, the interaction of PD-1 with PD-L-1 occurs in *trans* and produces inhibitory signals for attenuating T-cell responses, especially when these receptors are expressed on the T-cells of antigen presenting cells (APCs) or cancer cells. Antibodies against PD-1 and its ligands blocked these inhibitory signals (**Figure 3**). However, PD-1/PD-L-1 binding can also take place in *cis*, when the tumor cells or APCs express both PD-1 as well as PD-L-1 (36). Indeed, it was noticed that the PD-1 and PD-L-1 co-expressed on the same cancer cells or APC interact in *cis*. This, in turn, inhibits PD-L1’s ability to interact with T-cell intrinsic PD-1 in *trans* configuration, and reduces canonical PD-L-1/PD-1 inhibitory signaling. This was demonstrated through reconstitution and cell-culture assays. Failure of PD-1 inhibition to increase T-cell responses may be due to the *cis* PD-1/PD-L1 binding. Under these circumstances, PD-L-1 interacting to PD-1 expressed on T-cells to suppress signaling and cytotoxic responses, may be released by antibody binding to PD-1 expressed on tumor cells. Such an interaction may influence cancer immunotherapy, because simultaneous PD-1 and PD-L-1 suppression is required to overcome these conditions (12).

Despite interacting with PD-1, the ligand PD-L-1 was found to bind with B7-1 (CD80) protein and regulate the T-cell function. This interaction was extensively investigated through application of flow cytometry, ELISA and cell-to-cell bonding assays, and it was observed that PD-L-1-transfected cells interact to the PD-1-transfected cells; while, B7-1 cells bind to the CD28, and no interaction among PD-L-1 and B7-1 was noticed (37). When the same experiment was performed using purified protein rather than transfected cell lines, they found that only flexible PD-L-1 displayed strong interaction with B7-1, and that the soluble PD-1 and B7-1 compete for PD-L-1 interaction. It was concluded that the B7-1/PD-L-1 interaction occurs in *cis* configuration on the same cell, rather than *trans* between two cells. Such binding orientations may help to better understand these pathways and may be exploited in cancer chemotherapy. A similar study demonstrated that on APCs, PD-L-1 interacts to CD80 in *cis* and disrupts the PD-L-1/PD-1 binding in *trans* (38). Consequently, when a significant amount of CD80 is expressed by APCs, PD-L-1 is unable to engage PD-1 to diminish T-cell activation. In absence of the *cis*-PD-L-1/CD80 interaction, PD-1 greatly diminished autoimmune responses and tumor immunity. Subsequently, disruption of PD-1/PD-L-1 binding in *trans* is due to the *cis* PD-L1/B7-1 interaction on APCs (39). Through reconstitution assays and fluorescence readouts, they demonstrated that heterodimerization of ligands CD80 and PD-L-1 occurs only in *cis* and not in *trans*.

Additionally, tests utilizing cell biology and biochemistry demonstrated that *cis*-PD-L-1/CD80 contacts inhibit the CD80/CTLA-4 and PD-1/PD-L1 connections; yet CD80’s capacity to stimulate the CD28 a T-cell co-stimulatory receptor remains intact. Atezolizumab, a PD-L-1 blocking antibody (not PD-1 blocking) reduced CD80 expression on the cell surface of APCs; ipilimumab, an anti-CLTA-4 antibody, counteracted this effect by co-blocking the CTLA-4 axis. PD-L-1 produced immune-stimulatory impact by decreasing the CTLA-4 axis, which allowed anti-PD-L-1 and anti-CTLA-4 combination treatment to work in concert. These results indicated that in comparison to single agent treatment, combination immunotherapy targeting CTLA-4 and PD-1 or CTLA-4 and PD-L1 demonstrated stronger anti-tumor responses. The relationship between PD-L-2 and PD-1 may also be complex, and because there are reports of both co-stimulatory and co-inhibitory roles of PD-L-2, its influence on T-cell responses is debatable (40, 41).

It was discovered that CD4+ T-cell cytokine preparation and T-cell receptor-mediated proliferation were strongly suppressed by PD-1/PD-L-2 interactions. Additionally, the interactions between PD-1/PD-L-2 at low and high antigen concentrations were examined, and found that the former inhibited strong B7-CD28 signals, while the latter decreased cytokine production without impeding T-cell proliferation. Overall, it was concluded that PD-L-1 and PD-L-2 had overlapping functions, which indicated that this route was crucial in regulating T-cell responses. In addition to connecting with PD-1, the PD-L-2 also binds with repulsive guidance molecule-b (RGMb), a co-receptor for bone-morphogenetic proteins. It has been shown that the surfaces of natural T lymphocytes, macrophages, neutrophils, and dendritic cells express this co-receptor and regulates respiratory tolerances (42).

## 4 Therapeutic implication of PD-1/PD-L-1 blockage

In recent years, cancer immunotherapy achieved crucial breakthrough and revolutionized the therapy of advanced-stage cancer and in this case, immune-checkpoint blockade has been approved to be clinically relevant and remarkably successful in comparison to other immunotherapeutic approaches (43, 44). A considerable number of evidences suggested that the PD-1/PD-L-1 interactions in TME leads to suppression, exhaustion and apoptosis and create resistance from cytotoxic T-cell mediated killing of cancer cells. This increases the cancer cell proliferation and promotes survival, leading to cancer development and progression (45, 46). With these findings, PD-1/PD-L-1 pathway is considered as potential target for novel anticancer therapy.

For several human cancers, interfering PD-1 axis has been among the most efficient immunotherapies, and was revealed that PD-1/PD-L-1 inhibition increased anti-tumor T-cell activity through prevention or reversal of exhausted T-cells. This leads to an increased endogenous immunity in cancer patients and long-term antitumor response is produced against several cancer forms (47–50). However, till now, only mAbs as PD-1/PD-L-1 blocking agents have been approved for therapeutic applications in cancer immunotherapy. Although, these antibodies achieved exciting clinical successes on patients responding to these therapies, a sizable population of cancer patients failed respond properly to the current immune-checkpoint modulators and could not get benefited from antibody therapy. Primary adaptive and acquired resistance along with poor oral bioavailability, severe immune-related adverse effects on multiple organs, high cost and other issues restricted the application of these mAbs and the outcomes in this scenario strongly indicating the requirement for developing new immunotherapeutic agents. Accordingly, in recent years, the attention of scientific fraternity involved in drug discovery research has been diverted toward this issue and several novel SMIs that can target the PD-1/PD-L-1 axis in a way similar to the available mAbs to enhance the adaptive immune responses are being investigated. The reports in this regard indicated that the SMIs displayed potential cell inhibition activities and exhibited the ability to overcome the problems associated with mAbs (51, 52).

## 5 Antibodies as PD-1/PD-L1 inhibitors

Immune-checkpoint blocking antibodies are based on relatively novel therapeutic strategy which act by modulating T cell pathways regulating T cells. PD-1/PD-L-1 blocking antibodies found to suppress the tumor progression in mice through restoration of T cell cytotoxicity (5). This finding resulted in the recognition of PD-1/PD-L1 pathway as a new target for anticancer therapy and research for developing new therapeutic antibodies were immediately started. Consequently, in the last few decades, several of the PD-1/PD-L1 blocking mAbs were discovered, which are crucial part of cancer therapeutic arsenal and revived the antitumor immunotherapy. These antibodies successfully block the immunosuppressive activity triggered by PD-1/PD-L1 interaction. In this case, association of PD-1 protein expressed on T-cells with its PD-L-1 ligand is inhibited to reactivate the cytotoxic responses of T-cell against various tumor forms (53).

Till date a number of PD-1/PD-L-1 blocking mAbs have been approved by different Drug Regulatory Agencies worldwide for therapeutic application in cancer immunotherapy. Among the mAbs approved by the US FDA nivolumab (Opdivo) and pembrolizumab (Keytruda), are PD-1 blockers and recommended for treating metastatic NSCLC and melanoma, respectively in 2014 (54, 55) and atezolizumab (Tecentriq), a PD-L-1 inhibitor antibody was approved in 2016 for treating metastatic NSCLC and urothelial cancer (56). Avelumab (Bavencio) and durvalumab (Imfinzi) are two other anti-PD-L1 antibodies, received their first global approval in 2017 for treating metastatic urothelial carcinoma and Merkel carcinoma cell, respectively (57, 58). These antibodies were found to be effective against other cancer types and apart from their first approval, these antibodies were approved for treating other cancers as well. For example, currently, pembrolizumab approved in more than eighty countries for treating several cancers, including urothelial carcinoma, head and neck squamous cell carcinoma, primary mediastinal large B-cell lymphoma, Markel cell carcinoma, mismatch repair-deficient solid tumor, renal cell, cervical, hepatocellular and gastric cancers (59).

In conjunction with chemotherapy, atezolizumab and durvalumab were authorized by the US FDA in 2019 and 2020 respectively, as first-line treatment for advanced stage SCLC (60). The European Medicines Agency (EMA) AND US FDA authorized atezolizumab and bevacizumab together, which improved the overall patient survivals with unresectable hepato-cellular carcinoma (61, 62). Avelumab is used for treating renal cell carcinoma, whereas lung and colorectal malignancies can be treated with durvalumab and nivolumab, respectively (63). The anti-PD-1/PD-L-1 blocker antibodies approved so far along with the cancer types they are recommended are listed in **Table 1**.

**Table 1:** PD-1/PDL-1 blocking mAbs approved so far and their adverse effects.

Monoclonal Antibody	Approval Year	Type and Target of action	Approved for	Adverse effect	Reference
Pembrolizumab (Keytruda; Merck)	2014	An IgG4κ monoclonal antibody that has been fully humanized and binds to PD-1	urothelial carcinoma, metastatic melanoma, head and neck squamous cell carcinoma, NSCLC, solid tumors with low mismatch repair or high microsatellite instability, classical Hodgkin Lymphoma, cervical cancer, gastric cancer, large B-cell lymphoma, hepatocellular tumor, Markel cell carcinoma, renal cell cancer, and esophageal squamous cell cancer, endometrial carcinoma	Immune-related adverse events: pneumonia, encephalopathy, myocarditis, hepatitis, and colitis. Other common adverse effects: Pruritus, reduced appetite, fatigue, nausea, diarrhea, dyspepsia, rashes, pyrexia, musculoskeletal pain, constipation, cough and abdominal pain	(55, 59, 64)
Nivolumab (Opdivo; BMS)	2014	IgG4 monoclonal antibody that is fully humanized by genetic engineering and binds to PD-1	Renal cell carcinoma, metastatic melanoma, metastatic NSCLC, and metastatic SCLC, classical Hodgkin Lymphoma, urothelial carcinoma, hepatocellular tumor, head and neck squamous cell carcinoma, colorectal cancer	Immune-mediated colon inflammation, lungs, kidneys, and liver; hypothyroidism and hyperthyroidism. Autoimmune diabetes.	(54, 64)
Cemiplimab (Libtayo; Sanofi)	2019	Created by recombinant DNA technology, humanized IgG4 monoclonal antibody, binds to PD-1	Basal cell carcinoma, NSCLC, cutaneous squamous cell carcinoma,	Colitis, pneumonia, hepatitis, nephritis, renal failure, endocrine abnormalities, and skin responses that are immune-mediated. Others: rashes, fatigue, and diarrhea.	(64, 65)
Atezolizumab (Tecentriq; Genentech)	2016	IgG1 isotype monoclonal antibody with high affinity that has been developed and fully humanized; binds to PDL-1	Multiple cancers include triple negative breast cancer, extensive-stage SCLC, NSCLC, and metastatic non-squamous NSCLC.	Immune related reactions affecting multiple organs including liver, skin, lungs, colon, endocrine system etc. Other common adverse effects include urinary tract infection, fatigue, nausea, decreased appetite and dyspnea.	(56, 64)
Durvalumab (Imfinzi; Astra Zeneca)	2017	Fully natural human IgG1κ monoclonal antibody; binds on PDL-1	metastatic urothelial carcinoma, non-small cell lung cancer	Musculoskeletal pain, urinary tract infection, fatigue, nausea, constipation, reduced appetite, upper respiratory tract, rashes, coughing, dyspnea, peripheral edema, stomach discomfort, dehydration, and overall decline in health. Rare adverse effects are pneumonitis, arterial fibrillation, acute myocardial infarction, and diabetes	(57, 64)
Avelumab (Bavencio; Merck)	2017	High-selective, high affinity, fully humanized IgG1monoclonal antibody of IgG1 isotype; binds on PDL-1	Metastatic urothelial carcinoma, Merkel cell carcinoma, and renal cell cancer	Immune-mediated: colitis, pneumonia, hepatitis, adrenal insufficiency, diabetes, nephritis, and hyperthyroidism. Others: rashes, fatigue, nausea, diarrhea, discomfort in the musculoskeletal system, appetite loss, and urinary tract infections, peripheral edema and infusion related adverse effects.	(58, 64)
Dostarlimab (Jemperli; GSK)	2021		Primary advanced or recurrent endometrial cancer	Immune-mediated adverse effects: colitis, pneumonitis, hepatitis and endocrinopathies including hypothyroidism, nephrities with renal dysfunction and skin problems. Other adverse effects are diarrhea and hypertension.	(66)
Retifanlimab (ZYNYZ; Incyte Biosciences)	2023	PD-1	Metastatic or recurrent locally advanced Merkel cell carcinoma (MCC) in adults patients	Immune-mediated pneumonitis, colitis, hepatitis, endocrinopathies and renal dysfunction. May cause adrenal insufficiency, thyroid disorders	(67)

Despite tremendous success of anti-PD-1/PD-L-1 mAbs as immunotherapeutic agents for treating a variety of cancers, these agents suffer from noticeable drawbacks, indicating the need for other drugs to incorporate in the cancer immunotherapy. The first problem associated with the use of current mAbs, is that all the patients are not responsive to the treatment; furthermore, a small proportion of the responsive patients face relapse. In this regard upregulation of alternate checkpoints including TIM-3 and inflammatory factors in the TME are responsible. This results in the development of adaptive resistance by tumor cells (68, 69). The development of mAbs as targeted immunotherapy was also aimed to minimize the common adverse effects associated with the use of non-targeted anticancer therapies by increasing the selectivity and reducing the effect of drugs on the normal human cells. However, apart from unresponsiveness and the cases of relapse, the usage of anti-PD-1/PD-L1 mAbs are associated with a number of new undesirable effects with varying magnitude and frequencies. Clinical investigations revealed that the most common adverse effects caused by these antibodies are involving the organs such as endocrine glands, skin, lungs, and liver.

The lower tumor penetration, high cost of manufacturing and autoimmune side effect rates further restricted the use of anti-PD-1/PD-L-1 mAbs. The most frequent side effects linked to anti-PD-1 mAbs usage include thyroiditis and pneumonitis; while, hepatitis and immune-mediated colitis are the primary side effects linked to PD-L-1 blocking antibodies. On the whole, mean incidence and severity of adverse effects are greater with the antibodies targeting PD-1 protein as compared to those targeting the ligand PD-L1 (70, 71). Enhanced cytotoxic efficacy of T cells against normal human cells, increase in the level of pre-existing auto-antibodies and greater production of pro-inflammatory cytokines are some of the important explanation proposed for the harmful autoimmune incidences associated with the therapies blocking immune checkpoints (72).

## 6 Small molecule inhibitors (SMIs) of PD-1/PD-L-1 axis

Clinical success of the mAbs targeting immune-checkpoints provided a new direction to the medicinal chemists for developing of SMIs that can target PD-1/PD-L-1 axis in cancer treatment. However, there are certain obstacles including insufficient structural information, has limited their development. To overcome these obstacles, the available structural data of protein interactions especially those between ligands and receptors are being studied. The Protein Data Bank (PDB) can be assessed for crystal structures along with the pharmacological targets of PD-1 and PD-L and these structures also provide information on their binding interactions (73–75). As indicated by the data from several groups, the domain in these proteins lacks proper cavities to accommodate low molecular weight compounds. Consequently, efforts to develop small molecules as blockers of PD-1 and PD-L1 were not as successful as antibodies up to this point (76, 77). However, the recent investigations indicated the development of the SMIs as promising approach to get rid of the problems associated with current immunotherapy based on antibodies and expected to provide several benefits in the field of immune checkpoint inhibition.

Consequently, in recent time, considerable efforts are being made to develop SMIs as alternative anticancer therapy targeting PD-1/PD-L1 axis (78, 79). These molecules seem to be advantageous over the current mAbs immunotherapeutic agents, as they may offer short half-life effects beneficial in terms of immune-related adverse effects and elimination of immunogenicity problems, lower cost production, greater stability, greater tumor penetration and improved bioavailability leading to increased bio-efficiency (80, 81). Within TME, the SMIs displayed higher diffusion rate in comparison to antibodies and able to target PD-1 protein in the other cellular sources, which could avoid the macrophage-mediated resistance that is the case with therapies involving anti-PD-1 antibodies (82).

Despite these advantages, the success to develop small molecule targeting PD-1/PD-L-1 or any other immune-checkpoint receptors are still inconsiderable, and no molecule could reach the clinic so far. The design and development of SMIs of PD-1/PD-L1 has been challenging primarily due to restricted structural elucidation of the binding proteins; however, amazing progress is anticipated with the advancements in the structural characterization of these proteins. The recent developments indicated that targeting the larger, hydrophobic, and flat interaction interface PD-1/PD-L-1 axis without deep binding pockets is difficult. Bristol-Meyers Squibb (BMS) has been the first to develop and patent the non-peptide-based small molecules, commonly known as “BMS molecules”, could convincingly inhibit the interplay among PD-1 and PD-L1 (83); though, poor drug-like properties has been reported for these compounds. These molecules were demonstrated to block the PD-1/PD-L-1 binding and reinstated the cytotoxic activity of T-cells (84). Interaction of these SMIs to the PD-L-1 results in deep hydrophobic and cylindrical pocket formed by the interface of the two monomers. Indeed, the development of BMS compounds offered impeccable starting points for the rational structure-based approach for new drug development. The structures of some potential BMS compounds that showed promising results are depicted in **Supplementary Figure S1** (**Supplementary file**).

The BMS group was able to characterize the PD-L-1 targeting SMI compounds and suggested their mechanism using various techniques including X-ray crystal structure studies and NMR. They were able to delineate the direct binding of BMS compounds (BMS-8 and BMS-202) to PD-L-1 using NMR assay and crystal structure studies. It was shown that the compounds inhibited PD-1/PD-L-1 interaction by inducing PD-L-1 dimerization (84). Another mechanism identified for the BMS molecule BMS-1166 is partial and specific inhibition of the PD-L-1 glycosylation and functional inactivation of PD-L-1 by preventing its export to Golgi from the endoplasmic reticulum (ER) (85). Some biphenyl compounds have shown to exert their action via induction of a two-step internalization process. The PD-1/PD-L-1 axis is inhibited by these compounds by initiating the dimerization of PD-L-1 at the cell surface (86). The cis-interacting homodimers trigger a loss of PD-L-1 via rapid internalization into the cytosol, inhibiting its interaction with PD-1. Another compound CA-170 (1), being more polar molecule as compared to the BMS biphenyl compounds, is shown to bind directly to the PD-L-1 without interfering or disrupting the PD-1/PD-L-1 complex, thereby antagonizing it (87, 88). This mode was analogous to the reported anti-PD-1 antibodies which are known to antagonize the PD-1 signaling without disruption of the PD-1/PD-L-1 complexation.

## 7 Non-BMS small molecules PD-1/PD-L1 inhibitors

**Figure 4:**
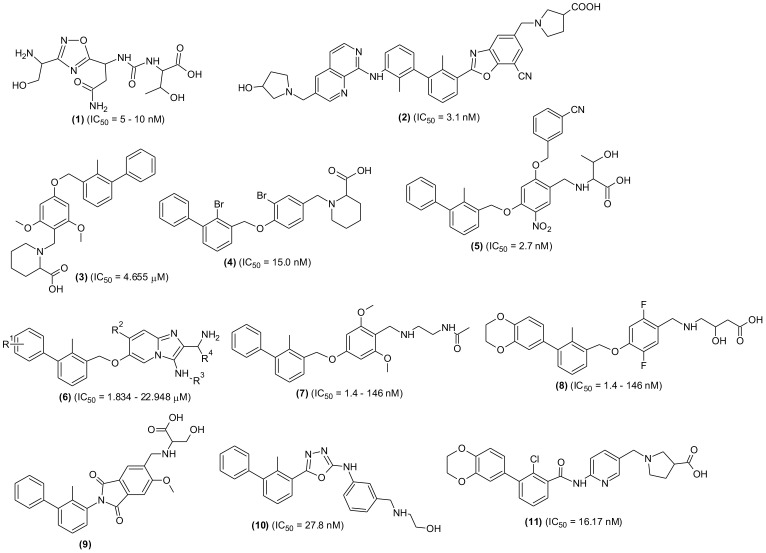
Chemical structures of newly designed small molecules-based PD-1/PD-L-1 interaction blockers as cancer immunotherapeutic agents.

Although, numerous SMIs targeting PD-L-1 are patented in recent years, only CA-170 (1) has entered the clinical trial so far. The compound was tested for treating lymphomas and advanced solid tumors (NCT02812875), MSI-H positive cancer, lung cancer, Hodgkin lymphomas and head-neck/oral cavity cancer. However, few research groups reported no direct binding of the molecule with the PD-L-1 target (89, 90). A new oral small molecule (INCB086550) (2) was identified and characterized that selectively inhibited PD-1/PD-L-1 interaction and induced PD-L-1 internalization and dimerization in *in vitro* models and increased production of cytokines in primary immune cells (91). In-line with the PD-1/PD-L-1 blockage, the compound activated T-cells, and reduced tumor development in CD34+ humanized mice *in vivo*. Furthermore, the compound’s PD-1/PD-L-1 blockage in peripheral blood cells with enhanced immune-activation and controlled tumor growth was validated by the preliminary findings of a phase I clinical investigation. The experimental results indicated the compound is one of the promising small molecule drugs and an alternative to antibody-based immunotherapy (**Figure 4**).

An inclusive structure-based virtual screening was accomplished using transdisciplinary approach to discover new non-BMS small molecular structures that can target PD-1/PD-L-1 interaction (51). Numerous new molecules from various drug repositories such as SPEC, Enamine, NCI, etc. were investigated using crystal structure of human-PD-L1 for molecular docking and identified compounds having the ability to stimulate cytotoxic function of T cells similar to anti-PD-1/PD-L1 antibodies; in this regard they used *in silico* investigation for hit identification followed by *in vitro*, *in vivo* and *ex vivo* investigations. Based on the results achieved, it was claimed that the new molecules could enable widespread penetration of T-cells into 3D solid tumor and recruit cytotoxic T-cells to TME *in vivo*. Using a structure-based virtual screening method, they screened almost 900,000 molecules from synthetic chemical libraries using a computationally driven methodology. This screening resulted the identification of ninety five virtual hits displaying high score values with good spatial fitting inside the PD-L-1 pocket and acceptable pharmacokinetic and toxicity profiles. Furthermore, using standard biochemical fluorescence-based binding assay, they identified 16 compounds as validated hits. The results of this study were comparable with BMS202 as positive control and predicted that the new validated molecules will interact with PD-L1 in the same way as BMS inhibitors. The stabilization effects seen in DSF and WaterLOGSY NMR experiments further supported the binding interaction of most promising compounds.

Another study reported extensive screening of a SMI, designated as PDI-1 (3) and reported to display significant anti-tumor potential through reduction of the PD-1/PD-L1-induced T-cell exhaustion in both *in vitro* as well as *in vivo* settings. They found that the test molecule could interact with murine and human-PD-1 and PD-L1 in competitive manner. The compound increased cytotoxic activity against *ex vivo-*activated T-cells for human melanoma and lung cancer cells with concomitant increase in the granzyme B, inflammatory cytokines, and perforin productions. Using Luciferase reporter assay, the researchers demonstrated an increase in the TCR-mediated activation of NFAT in PD-1/PD-L-1. Moreover, intraperitoneal injection of the compound in a syngeneic mouse tumor model led to decreased tumor growth in human PD-L-1 transfected murine melanoma and lung cancer, enhanced tumor infiltrating CD8+ cell abundance, and reduced tumor and FoxP3+ CD4+ cell abundance expressing PD-L-1. The anticancer activity displayed by the test compound was comparable to atezolizumab, an existing immunotherapeutic antibody (92).

A cost-effective and less time-consuming synthesis and screening of di-bromo-based SMIs targeting PD-1/PD-L-1 was reported previously (89). Final molecule (4), a PD-L-1 antagonist, dissociated the PD-1/PD-L-1 complex, restored cancer-reactive T-cells activation and incited adaptive immunity similar to anti-PD-L-1 antibodies. Being a small molecule, the final compound was nontoxic even at high concentration; in addition, it was cheaper to prepare and probably non-immunogenic. Antagonist induced dissociation NMR assay (weak-AIDA-NMR), a novel methodology was used to design the scaffolds of the series, validated with Homogenous Time-Resolved Fluorescence Affinity technique, and further confirmed by cell-based assay. The prepared derivative was claimed to be the strongest PD-L1 inhibitor among the small molecules reported so far, including the potent ones from BMS series such as BMS-1166 and BMR-1001.

Two series of new compounds consisting of biphenyl linked to nitrophenyl moiety through methoxy group as core structural moiety were prepared and screened for PD-1/PD-L-1 inhibition efficacies. All the compounds were reported to have remarkable inhibitory effects (IC_50_ = 2.7–87.4 nM); however, compound (5) with hydrophilic region attached to the central nitrophenyl moiety was found to have the highest activity. In Lewis lung carcinoma, the selected compound interacted with PD-L-1 with no toxicity and an enhanced secretion of IFN-γ was observed in a dose-dependent manner. Using immunohistochemistry and cytometry assay it was revealed that the compound (5) countered PD-1-induced immune-suppression in the TME and stimulated antitumor immune responses. Further investigations have shown that the compound displayed greater *in vivo* anticancer activity than BMS1018 (93).

A new structural scaffold containing imidazopyridines blockers was identified. The molecular design was performed based on the previously solved high-resolution structures that bound to PD-L-1 (94). In this study, Groebke-Blackburn-Bienayme multicomponent reaction (GBB-3CR) was used to prepare a number of imidazo [1,2-α] pyridine compounds and evaluated through biophysical assays. The PD-L-1 co-crystal structure indicated the interplay of the molecules with PD-L-1 and the potential leads with low-micromolar PD-L-1 affinities were identified (6). The study was claimed to be a door-opening for new bioactive scaffolds as PD-L-1 blockers and in their previous investigations, the same research group developed small molecules that showed positive results as PD-L-1 inhibitors. In this study, they reported two new classes of macrocyclic peptides as PD-1/PD-L-1 pathway blockers. Using crystal structures they showed direct binding with PD-L-1 and proved that the molecules antagonized PD-L-1 signaling and restored T-cell functions similar to antibodies (95). In another investigation, they performed X-ray and NMR characterization of two classes of similar inhibitors including [3-(2,3-dihydro-1,4-benzodioxin-6-yl)-2-methyphenyl]-methanol and (2-methyl-3-biphenylyl)-methanol and analogues (7, 8). The X-ray structures of the protein and drug complex showed that the first inhibitor induced an engorged interaction interface which resulted in the open face-back tunnel via the PD-L-1 dimer, while the second one capped on one side of the channel (96).

Recently, several biphenyl analogues were synthesized and their *in vitro* evaluation as PD-L1 blockers was performed (97). In an *in vitro* experiment, the most promising molecule (9), effectively improved the cytotoxic potential of peripheral blood mononuclear cells (PBMCs). The molecule showed PD-1/PD-L-1 inhibition, induced PD-L-1 dimerization and subsequent internalization and improvement in its localization to endoplasmic reticulum (ER). The molecule suppressed tumor growth in the *in vivo* lung and colorectal cancer (CRC) models. Consistent with the PD-1/PD-L-1 pathway inhibition, the molecule induced T-cell stimulation and reversed the inhibitory TME. A small molecule biphenyl oxadiazole derivative (10)was identified as bifunctional inhibitor and it was discovered that the compound promoted internalization of PD-L-1 from cell surface to inside the cytosol and subsequent degradation in cancer cells via lysosome-dependent pathway (98). In an *in vivo* model, the selected molecule inhibited tumor growth through activation of antitumor immunity. In search of effective SMIs of PD-/PD-L1 interaction, a series of benzamide analogues was synthesized, and screened. The most potent compound of the series, a benzamidopyridinyl-pyrrolidine carboxylic acid (11) effectively activated the anticancer immunity of T-cells in the PBMC killing assay. The IC_50_ value for the compound was calculated as 16.17 nM and performed superior activity than BMS202. Docking analysis of the compound was conducted to evaluate the binding mode, and results indicated the compound as promising lead for further screening (52).

**Figure 5:**
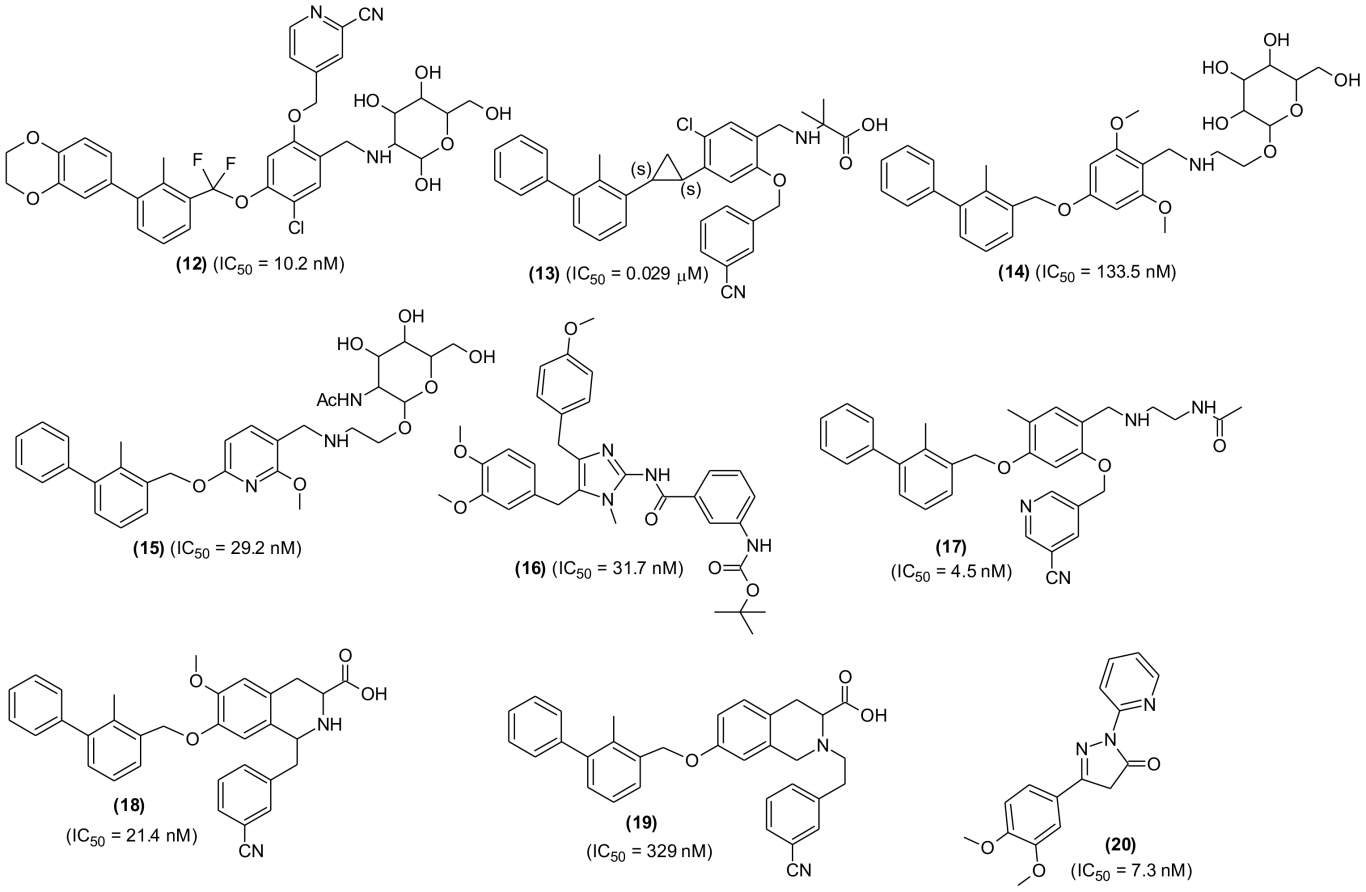
Chemical structures of potential small molecules as PD-1/PD-L-1 interaction inhibitors.

Another series of novel analogues was designed through structural fine tuning of potent PD-/PD-L1 interaction blocker BMS-1002 along with alteration in the solvent interaction area and synthesized (99). The newly prepared molecules were characterized by difluoro methyleneoxy linkage instead of methoxy in BMS-1002. The compound with mannosamine tail (12) was observed to be most potent molecule in HTRF assay (IC_50_ = 10.2 nM). The compound remarkably stimulated the CD8+ T-cells activation, deferred tumor growth and displayed good tolerance in mouse model. The compound was considered a promising candidate for further investigations by the research team (**Figure 5**). In a similar experiment, a series of new derivatives possessing cyclopropyl moiety in place of methoxy linkage of BMS202 were designed, prepared, and screened for PD-1/PD-L-1 interaction blocking property. The most active derivative (13), a (*1S*, *2S*) isomer exhibited potent inhibitory action with 0.029 μM IC_50_ value and a selective PD-L-1 binding affinity (K_D_ = 1.554 × 10^-1^ μM). Moreover, in a dose-dependent manner, the compound was able to decrease H460 cell survival. The compound showed acceptable pharmacokinetic behaviors with an oral bioavailability of 21.58%, good metabolic stability, non-observable side effects and remarkable antitumor potential in LLC1 lung carcinoma model. Enzyme-linked immunosorbent and flow cytometry assay results showed that the compound suppressed tumor growth through activation of immune microenvironment (100).

Recently, a new strategy was described to improve the therapeutic effects of small molecules-based PD-L-1 antagonist, where the sugar-motifs were introduced to the structure via glycoside linkage (101). It was found that mannose or N-acetylglucosamine glycosides of small molecules displayed remarkably high activity in the INF-γ secretion. Among the tested glucoside compounds, two compounds (14, 15) demonstrated remarkable antitumor activity in the B16-F10 and CT26 tumor models with good *in vivo* tolerance. Moreover, through tumor-infiltrating lymphocyte analysis, they enhanced the levels of granzyme B+, CD3+, CD4+, and CD8+ T-cells post treatment with the glycosidic compounds. In another recent study, a research team from China synthesized naamidine J, a marine-based natural compound and its analogues through structural modification guided by their tumor immunological activities. Among the derivatives, the compound (16) suppressed PD-L-1 expression in RKO cells with lesser toxic effects. The compound displayed antitumor potential in MC_3_8 tumor bearing C_57_BL/6 mice through reduction of PD-L-1 expression and enhancement of tumor infiltrating T-cell immune response. The study provided a direction for new leads discovery from marine products as tumor immunological agents (102).

The biphenyl-bases of small molecules were extensively reviewed and their possible interaction with numerous amino acids present at the binding site were described (103). The Met115, Tyr56, Ala121 and Asp122 were identified as important targets for PD-1/PD-L-1 inhibition. Further investigations indicated that binding pocket in PD-L-1 receptor was hydrophobic and tunnel-shaped. Based on the binding interaction, SAR of the biphenyl-based SMIs was established. In a similar study, a team of researchers designed a series 1,2,3,4-tetrahydroisoquinoline - 3 - carboxylic acid analogues as PD-1/PD-L-1 inhibitors. They cyclized benzylamine moiety to the ether-linkage of the 5-cyano-3-pyridinylmethoxy portion of their own previously evaluated compound LH1305 (IC_50_ = 4.5 ± 0.2 nM) (17). Among the new derivatives, the compound containing cyanophenyl or 5-cyano-3-pyridinyl moieties (18) at 1- position exhibited remarkable inhibition (IC_50_ = 21.4 ± 0.6 nM), which regarded as a future lead molecule for further study; while the compounds with direct linkage to the nitrogen of the scaffold (19) displayed lower inhibition (IC_50_ = 329 ± 47 nM) in protein-protein interaction (PPI) assay (104). A series of new compounds possessing pyrazolone moiety demonstrating nanomolar anti-PD-L-1 activity was characterized (105). Several synthesized derivatives showed excellent affinity to PD-L-1, blocked its interaction and inhibited the recruitment of Src-homology region-2 domain containing phosphate to PD-1. They identified five most active inhibitors among the series, one of them is compound (20). Based on *in vitro* screening data, SAR was established, and pharmacokinetic properties were evaluated. The SAR study revealed that the phenyl-pyrazolone moiety could provide a new approach in designing PD-L1 blocking agents, which night be useful to effectively overcome cancers and other pathological conditions associated with PD-1/PD-L-1 checkpoint.

**Figure 6:**
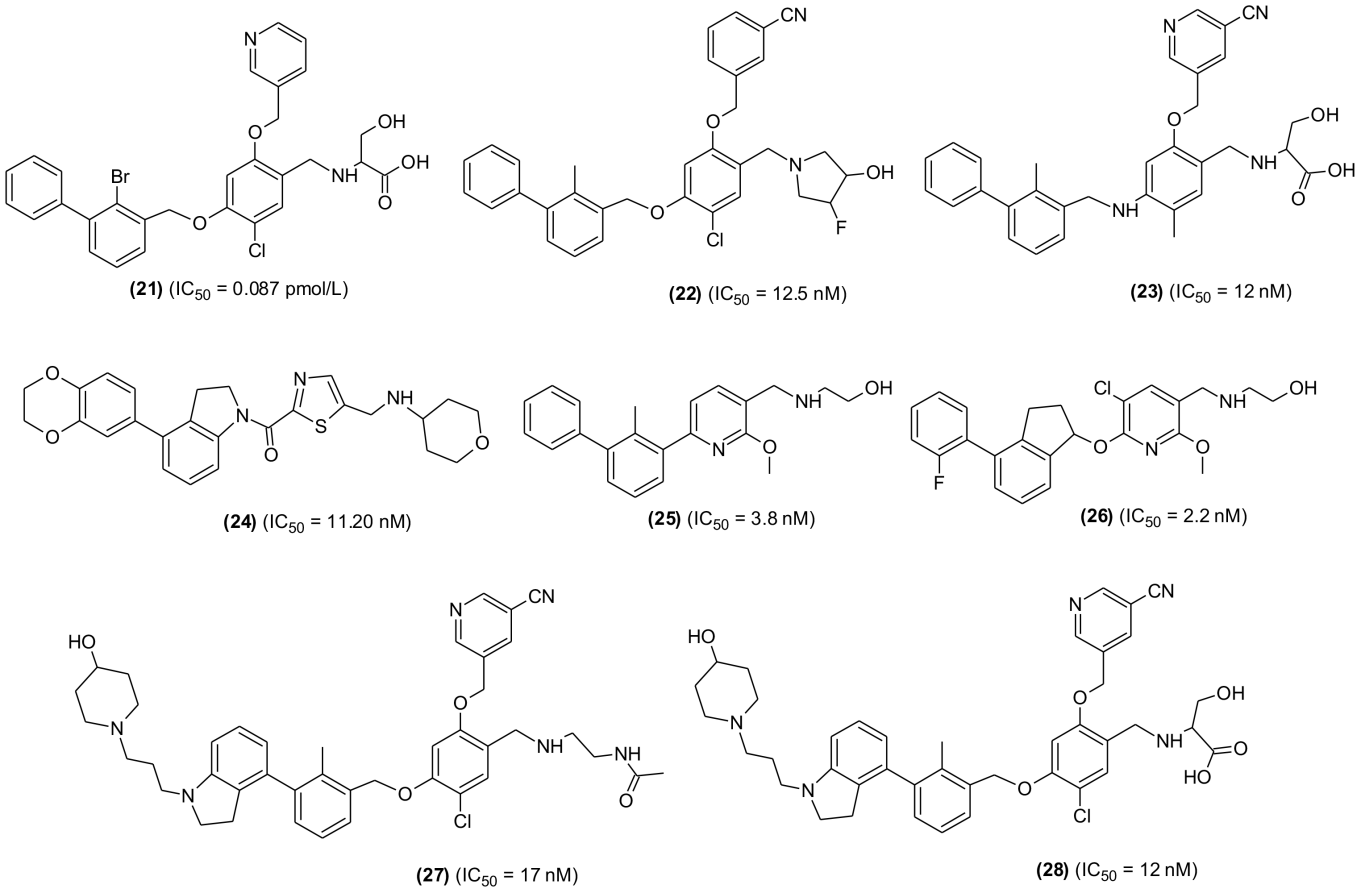
Representative chemical structures of new small molecules-based PD-1/PD-L-1 inhibitors for cancer immunotherapy.

Another SMI, YPD-29B (21), suitable for oral delivery was also developed and tested. The compound selectively and potently inhibited binding between PD-1 and PD-L-1, which was based the induction of PD-L-1 internalization and dimerization, T-lymphocyte activation and consequently could overcome the immunity tolerance in the *in vitro* setting. Furthermore, YPD-30, which is a prodrug of YPD-29B, was developed to improve its druggability, displayed remarkable antitumor activity with good *in vivo* tolerance in humanized mouse model. Based on results, YPD-30 was considered a future drug candidate for cancer immunotherapy (106). In an extensive study, PD-1/PD-L-1 pathway blocker compounds with resorcinol dibenzyl ether scaffold were designed and developed (107) (**Figure 6**). The most active compound (22) displayed significant inhibition in homogenous time-resolved fluorescence binding (HTRF) assay (IC_50_ = 12.5 nM) and increased IFN-γ production in a co-culture model of Hep3B/OS-8/h-PD-L-1 and H22 hepatoma tumor. In H&E staining and flow cytometric analysis the compound displayed antitumor effects through activation of immune TME. The tested compound may be considered as promising lead molecules for designing new PD-1/PD-L-1 blockers. Another promising lead compound (23) targeting hPDL-1 was developed and screened in *in vivo* and *in vitro* experiments. The compound is an aliphatic amine-linked tri-aryl derivative with highest binding affinity with hPDL-1 (IC_50_ = 12 nM and KD = 16.2 pM) and inhibited the hPD-1/hPD-L1 interaction in a competitive manner with approximately 2000-folds increased binding potency in comparison to hPD-1. The compound restored T cell function and increased the IFN-γ secretion. Furthermore, the compound remarkably suppressed tumor growth with no signs of toxicity and displayed optimum pharmacokinetic properties when administered through intravenous (iv) injection (108). A new series of 4-arylindoleine with thiazole moiety were developed and screened as PD-1/PD-L-1 axis modulators. Analogue (24) was identified as promising small molecule exhibiting remarkable activity (IC_50_ = 11.20 nM) in the HTRF assay. The reported compound suppressed the T-cell multiplication and promoted IFN-γ release (109). Furthermore, the compound displayed significant antitumor property in murine 4T1 breast carcinoma and CT26 colon carcinoma *in vivo* models. The flow-cytometry assay indicated that the suppression of tumor growth was through activation of immune microenvironment.

From a series of new biphenyl pyridine analogues, a small molecule (25) as PD-1/PD-L-1 blocking agent (IC_50_ = 3.8 ± 0.3 nM) was identified. The compound could enhance tumor cell killing through immune cell activation in CT26 mouse model with good pharmacokinetic properties. The immunohistochemistry and flow cytometry assay results showed that the compound was activating immune responses in TME, and it could act as a promising lead in developing potential SMIs for PD-1/PD-L-1 axis (110). A similar research reported the synthesis and SAR investigation of 31 indane compounds as novel PD-1/PDL-1 SMIs (111). The SAR study indicated that, (S)-indanes demonstrated greater potency with a compound shown in structure (26) displayed highest potency (IC_50_ = 2.2 nM). In a cell-based assay, the compound was shown to induce immune response of PBMCs toward MDA-MB-231 cells and restored the T-cell function through increased IFN-γ secretion. Synthesis and screening of new 4-phenylindoline derivatives by time-resolved fluorescence assay led to the identification of two potent PD-1/PD-L-1 SMIs (27 and 28) (IC_50_ = 17 and 12 nM, respectively). Molecular simulation and docking analysis of compounds (28) suggested that the potent inhibitory activity was due to interaction of N-atom present in the indoline side chain fragment with the amino acids present in the binding site of PD-L-1. Moreover, the compound (27) showed remarkable inhibition of PD-1/PD-L-1 interaction in a co-culture model of PD-1, expressing Jurkat cells and the PDL-1/TCR activator-expressing CHO cells (112).

## 8 Bifunctional small molecules

**Figure 7:**
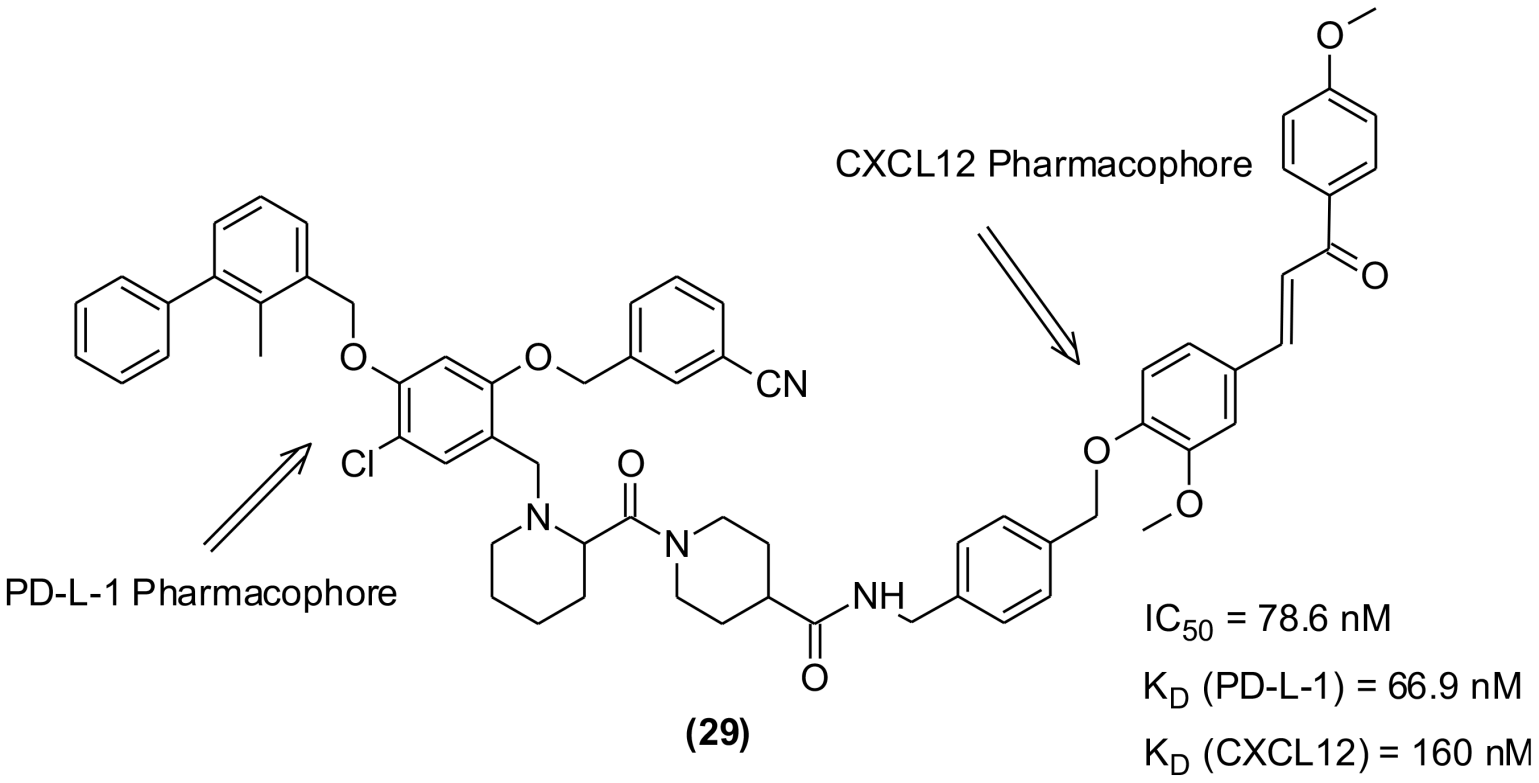
Bifunctional/dual targeting immunotherapeutic small molecules with pharmacophores of PD-L-1 and CXCL12 inhibitors.

To overcome the lower response rate of PD-1/PD-L-1 blocking immunotherapy among the patients, the researchers tried combinations of PD-1/PD-L-1 blocking agents with different antitumor medications. Few studies indicated synergistic effects and superior antitumor efficacy of combination immunotherapy with CXCL12 inhibitors than that demonstrated by monotherapy. However, unpredictable PK/PD properties as well as overlapping toxicities were observed (113). Further attempts were made to develop an alternative to combination therapy and focused on the use of a single drug exhibiting dual or multiple targeting properties because the PK/PD properties of single compound is more predictable in addition to having low chances of toxicity. Consequently, in recent years bispecific antibodies (bsAbs) with dual targeting capability gained attention and anti-PD-1/LAG-3, anti-PD-L-1/CTLA-4, and anti-PD-L-1/TGF-β bsAbs succeeded to clinical trials for dual immunotherapeutic anticancer agents. However, the problems associated with immunogenic side effects and poor pharmacokinetic performance remained unchanged. The discovery of new PD-1/PD-L-1 SMIs-based bifunctional/dual targeting immunotherapeutics are the new hope that may combat the drawbacks of antibodies. In this regard, a set of 21 bifunctional compounds were designed to target PD-L1 and CXCL12, simultaneously (114). The compounds were synthesized and bio-evaluated on both the targets. The pharmacophores were present as heads, while the tail and -OH group of the PD-L-1 and CXCL12 blockers, respectively were exposed to solvent and used to conjugate the two inhibitors through a linker (**Figure 7**). The compound (29) exhibiting highest inhibitory effect on human PD-L1 (IC_50_ = 78.6 nM, HTRF assay; KD = 66.9 nM) was identified. It also displayed significant binding affinity to CXCL12 (KD = 160 nM, SPR).

## 9 Future perspectives and conclusions

The cancer therapy has been revolutionized by the clinically successful immune checkpoint modulators. Among the immune check points, PD-1/PD-L-1 axis has garnered much attention as the potential target for treatment of different types of cancers. PD-1/PD-L-1 interaction results in the suppression of tumor inhibiting capability of T-cells. This information stimulated tremendous research interest to develop PD-1/PD-L-1 interaction blockers that may produce effective antitumor immune response against various cancer forms with low toxicity. It was observed that the blockage of PD-1/PD-L1 binding overturned the exhausted T-cells leading to efficient killing of cancer cells and displayed tremendous success in cancer immunotherapy of various melanomas, breast, and lung cancers etc. The antibodies provided unprecedented results, which led to the accelerated approvals by the regulatory organizations for several cancers formerly considered as lethal. In recent years, this approach has provided encouraging results that were also reflected in the clinical settings. Currently approved PD-1/PD-L-1 inhibitors for clinical applications with great success are humanized mAbs; however, their use has been restricted due to certain drawbacks such as poor bioavailability, high cost and complicated processing, and immunogenic adverse reactions.

On the contrary, the SMIs directly blocking the PD-1/PD-L-1 interaction exhibited promising results without immunogenicity, but their development is still in the initial phase. They attracted considerable research interest in the last decade. The small molecules are expected to be great drug candidates as well as promising tools to enhance the clinical outcomes in cancer immunotherapy. Besides showing good PD-1/PDL-1 interaction inhibition and lack of immune related adverse effects, the small molecules showed better tumor penetration, amenable oral administration, greater stability and are less expensive to prepare which make them easily accessible. Recently, various non-antibody SMIs have been investigated and several patents were granted to some researcher groups, including the one termed as BMS molecules disclosed by Bristol-Meyers Squibb; however, more detailed information about their activity is warranted. The BMS molecules were found to efficiently interact to hPD-L-1, inhibiting its binding with PD-1 and restored the T-cell activity. At present, there is an urgent need of developing new, safe, and effective PD-1/PDL-1 checkpoint inhibitors based on small molecular structures. Although, several structural scaffolds are being explored by the researchers for developing potential SMIs, biphenyl-based scaffolds are the most investigated ones showing promising results.

The development of mAbs for cancer immunotherapy is significantly ahead to that of SMIs because the highly hydrophobic, large, and flat PD-1/PD-L-1 interface is challenging to target. To achieve reliable measurements, it is necessary to measure the binding parameters for SMIs and PD-1/PD-L-1 interactions at both interactive and non-interactive interfaces. To accomplish this, complementary assays such as biochemical, biophysical, and cell-based techniques must be applied correctly. Over the last decade, there has been a steep rise in the investigations aiming to develop the bioassays for screening small compounds against PD-1/PD-L-1. This is due to our growing comprehension of the underlying mechanisms of these interactions. Strong instruments for determining the binding characteristics between the receptor and hits are based on biophysical and biochemical experiments; while the bioassays including alphaLISA, ELISA, T-cell-based assays, bioluminescent reporter cell-based assays are important for evaluating biological functions and in the elimination of false positive hits. The combinations of these approaches are extremely important to avoid false “hits” that may have binding capability with no PD-1/PD-L-1 blocking effects. These approaches opened an exciting possibility for development of safe and effective and small molecule-based PD-1/PD-L-1 blockers as anticancer immunotherapeutics.

## Author contributions

SJ: Conceptualization, Writing – original draft. AN: Funding acquisition, Writing – review & editing. WA: Software, Writing – original draft. KZ: Supervision, Writing – original draft.

## Supplementary material

Supplementary Figure 1 Chemical structures of BMS molecules being investigated as PD-1/PD-L-1 interaction inhibitors and considered as potential molecules for cancer immunotherapy.
