# Supplementary material for: Targeting PD-1/PD-L-1 immune checkpoint inhibition for cancer immunotherapy: success and challenges
Source: Front Immunol. 2024 Apr 10;15:1383456. doi: 10.3389/fimmu.2024.1383456 (PMC11039846; doi:10.3389/fimmu.2024.1383456)
Supplement: Supplementary Figure 1 — Chemical structures of BMS molecules being investigated as PD-1/PD-L-1 interaction inhibitors and considered as potential molecules for cancer immunotherapy. [file DataSheet_1.docx]

**SUPPLEMENTARY FIGURE**

**FIGURE S1**

**Figure S1:** Chemical structures of BMS molecules being investigated as PD-1/PD-L-1 interaction inhibitors and considered as potential molecules for cancer immunotherapy.
